# Systematic and general method for quantifying localization in microscopy images

**DOI:** 10.1242/bio.019893

**Published:** 2016-12-09

**Authors:** Huanjie Sheng, Weston Stauffer, Han N. Lim

**Affiliations:** Department of Integrative Biology, University of California Berkeley, 3040 Valley Life Sciences Building MC3140, Berkeley, CA 94720-3140, USA

**Keywords:** Manders' colocalization coefficient, Pearson's correlation coefficient, Co-occurrence, Colocalization, Image analysis, Microscopy

## Abstract

Quantifying the localization of molecules with respect to other molecules, cell structures and intracellular regions is essential to understanding their regulation and actions. However, measuring localization from microscopy images is often difficult with existing metrics. Here, we evaluate a metric for quantifying localization termed the threshold overlap score (TOS), and show it is simple to calculate, easy to interpret, able to be used to systematically characterize localization patterns, and generally applicable. TOS is calculated by: (i) measuring the overlap of pixels that are above the intensity thresholds for two signals; (ii) determining whether the overlap is more, less, or the same as expected by chance, i.e. colocalization, anti-colocalization, or non-colocalization; and (iii) rescaling to allow comparison at different thresholds. The above is repeated at multiple threshold combinations to generate a TOS matrix to systematically characterize the relationship between localization and signal intensities. TOS matrices were used to identify and distinguish localization patterns of different proteins in various simulations, cell types and organisms with greater specificity and sensitivity than common metrics. For all the above reasons, TOS is an excellent first line metric, particularly for cells with mixed localization patterns.

## Abstract (precis)

**Summary:** We evaluate a metric for quantifying localization in microscopy images, termed the threshold overlap score, which is simple to calculate and interpret, generally applicable, and suitable for mixed localization patterns.

## INTRODUCTION

Quantifying the localization of proteins, RNAs and complexes within cells can help determine their regulation and sites of action (Bolte and Cordelieres, 2006; Dunn et al., 2011; Zinchuk et al., 2007). Therefore, the development and evaluation of metrics to quantify localization is an important and shared goal of many different disciplines. Three common approaches to quantifying localization are: (i) measuring the overlapping fraction of two signals (Bolte and Cordelieres, 2006; Cordelieres and Bolte, 2014); (ii) measuring the correlation or rank order correlation of pixel intensities for two signals (Bolte and Cordelieres, 2006; French et al., 2008); and (iii) identifying objects and determining their fractional overlap or the distance separating them (Cordelieres and Bolte, 2014; Lagache et al., 2015). These metrics and less common alternatives (Zinchuk et al., 2007; Cordelieres and Bolte, 2014) have been successfully used in many applications. However, there are also many types of images and samples where the above metrics do not perform well and their results are difficult to interpret (Cordelieres and Bolte, 2014; Dunn et al., 2011; McDonald and Dunn, 2013; Wu et al., 2010), inconsistent (Dunn et al., 2011), and/or susceptible to arbitrariness and bias (Wu et al., 2010).

Metrics often encounter difficulty when images and samples have: a signal of low intensity compared to background and non-specific signals (Landmann, 2002; Dunn et al., 2011), a large proportion of pixels with background or non-specific signals (Dunn et al., 2011; Barlow et al., 2010), a nonlinear relationship between two signals (Cordelieres and Bolte, 2014), or mixed patterns of localization. Other important barriers to the use of some metrics include: limited testing (and consequently researchers are uncertain if the metric is suitable for their samples and application), underlying assumptions that limit their general application, and the need for customization or simulations that require specialized knowledge and skills. All these issues are common, resulting in researchers in many disciplines resorting to qualitative (and often inaccurate) assessments of localization by simply superimposing (or ‘merging’) images (Bolte and Cordelieres, 2006; Dunn et al., 2011). No single metric or analysis protocol will meet all requirements for all researchers (Bolte and Cordelieres, 2006; Dunn et al., 2011), but clearly additional tools to quantify localization are needed.

In this study we evaluated a metric for localization termed the threshold overlap score (TOS), which measures the overlap in signals above threshold intensity values. We use ‘localization’ and ‘localization pattern’ to refer to the measurement of overlap. If the overlap is greater than, less than, or the same as expected by chance then localization is categorized as ‘colocalization’, ‘anti-colocalization’ or ‘non-colocalization’, respectively. The first part of the study derives TOS and then describes a strategy of using it at many combinations of thresholds to generate a TOS matrix that can identify and distinguish features in mixed patterns of localization. The second part of the study applies TOS analysis to simulated data and experimental data obtained from public image repositories. The latter showed that values from the TOS matrix can distinguish the localization patterns of different proteins for a variety of cell types and organisms, and that they can do so with greater specificity and sensitivity than common metrics (Pearson's correlation coefficient, Manders' colocalization coefficients, and Spearman's rank correlation coefficient).

## RESULTS

### Calculating the threshold overlap score (TOS)

**Fig. 1.**
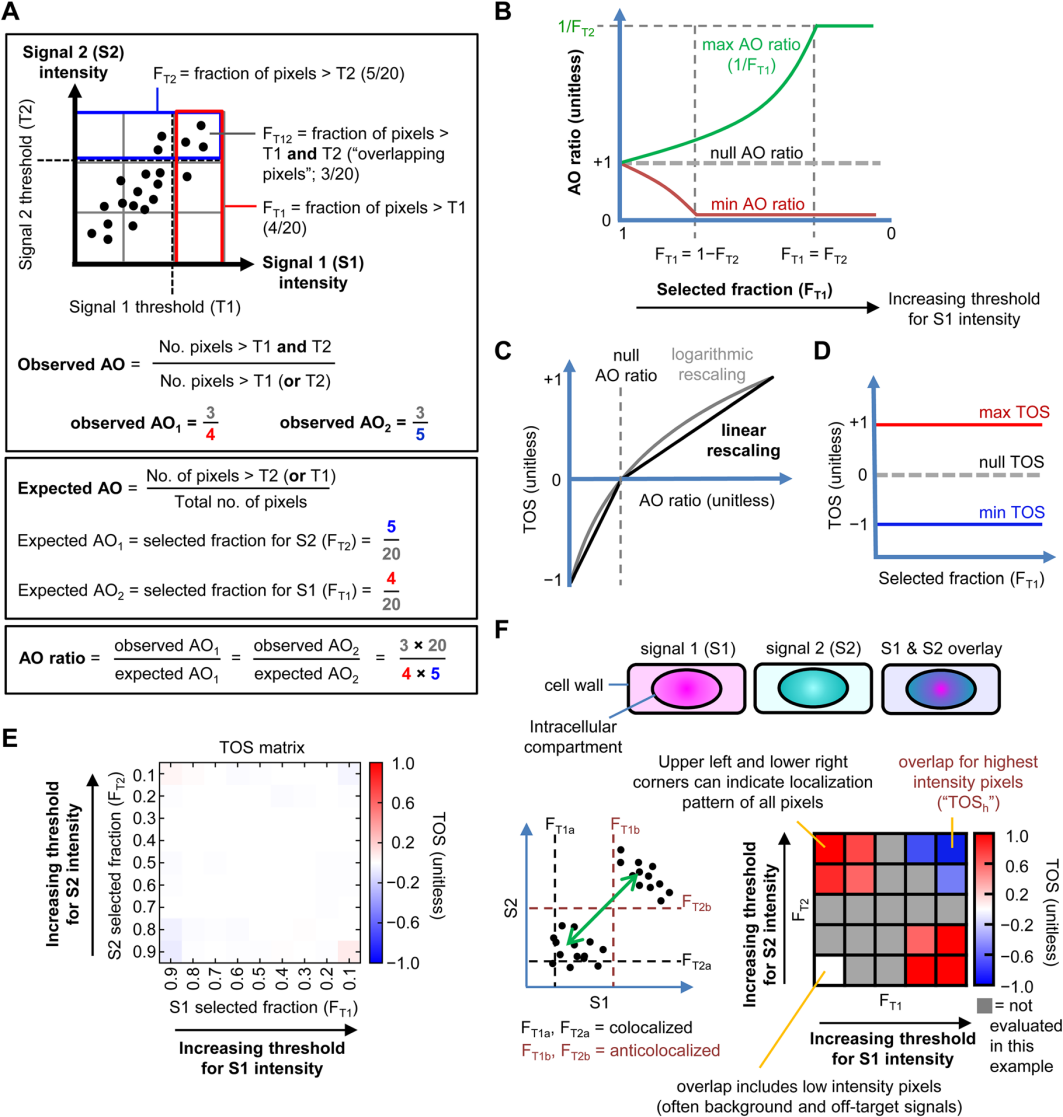
**Calculating the threshold overlap score (TOS) and generating TOS matrices.** (A) Calculation of observed AO, expected AO, and AO ratio. Thresholds are measured by the fraction of pixels with highest intensity in the cell, i.e. the ‘selected fraction’, which are F_T1_ and F_T2_ for signal 1 and 2, as explained in the main text. (B) Diagram showing maximum, minimum and expected AO ratios as a function of the threshold for signal 1, i.e. F_T1_ is varied and F_T2_ is fixed. Note: expected AO ratio is for the null distribution. (C,D) Threshold overlap score (TOS) is obtained from the AO ratio by rescaling linearly (or logarithmically) so the maximum, minimum and null values are +1, −1 and 0 for all selected fractions. (E) TOS matrix generated by simulating a uniform distribution for all threshold combinations (*n*=500 for each selected fraction). As predicted, the observed AO values for the simulated uniform distribution are close to the expected AO values therefore the AO ratio is ≈1 and TOS is ≈0 at all threshold combinations. (F) Thresholds can affect quantification and characterization of localization. Hypothetical cells with mixed intracellular localization patterns for two signals (S1 and S2). Cells have uncorrelated off-target signals and negatively correlated on-target signal. Note: although the off-target signals appear uniform, the signals have variation as shown in the scatterplots. Scatterplot to the left shows that the determination of the localization pattern depends on the threshold selected. Thresholds at low signal intensities (F_T1a_ and F_T2a_) will measure localization of both off-target and on-target signals and together they have a net positive correlation as shown by the green arrow (i.e. colocalization). Thresholds at high intensities (F_T1b_ and F_T2b_) will measure localization of only the on-target signal, which has a negative correlation (i.e. anti-colocalization).

The first step in calculating TOS is measuring the observed fraction of pixels that exceed the threshold of one signal that also exceed the threshold of a second signal (Fig. 1A). That is, measuring the ‘fractional area of overlap’ (abbreviated to ‘AO’). Instead of choosing thresholds by selecting specific values for the intensities, which in turn specify fractions of pixels for signals 1 and 2 (F_T1_ and F_T2_, respectively), we directly chose these fractions (following rank ordering of the pixels by intensity). This approach of specifying thresholds in terms of selected fractions rather than as values allows observed data from individual cells that have different intensities and total numbers of pixels to be more easily combined.

Therefore,

(1)
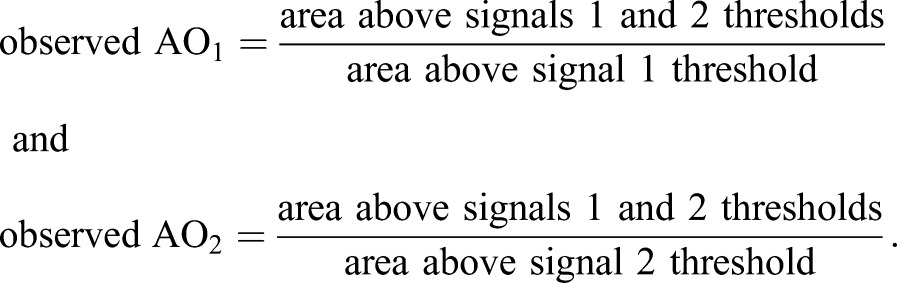


The second step is normalizing the observed AO_1_ and AO_2_ by the expected AO_1_ and AO_2_ (for uniformly distributed random signals), which are equal to F_T2_ and F_T1_ respectively, resulting in the AO ratio (Fig. 1A). Because it may seem counterintuitive that AO_1_ and AO_2_ are normalized by the threshold of the other signal, we consider the example of a cell with 100 pixels and selected fractions for signal 1 and 2 of 50% (F_T1_=0.5) and 10% (F_T2_=0.1), respectively. In this example, 50 and 10 pixels are selected for signals 1 and 2, respectively. If the selected pixels for signal 1 are uniformly distributed throughout the cell then half of them would be expected to overlay the selected pixels for signal 2 (irrespective of their distribution), which is 5 pixels. For the selected pixels of signal 1, this expected 5 pixel overlap represents 0.1 of them (i.e. 5 out of 50), which is equal to the selected fraction for signal 2 (F_T2_) as stated above. This normalization assumes a null distribution with pixel intensity values uniformly distributed across the cell and independent. Note: the point spread function with autocorrelation between pixels does not alter the predicted value but it does affect its variance (Dunn et al., 2011). From Eqn 1,
(2)
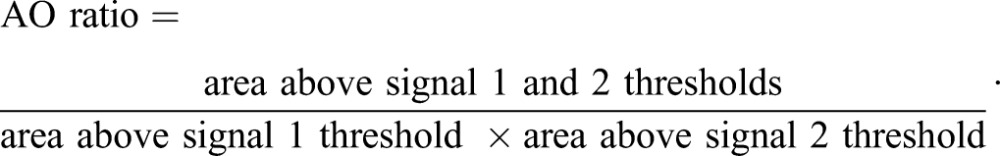
The AO ratio has the same value when calculated from the observed AO_1_ or AO_2_. The AO ratio ≈1, >1 or <1, when the pixels above the threshold of each signal overlap by the same, greater than, or less than the null distribution. The minimum AO ratio depends on F_T1_+F_T2_ (Fig. 1B). If F_T1_+F_T2_ ≤1, the minimum AO ratio can be zero because it is possible for the selected pixels for each signal to not overlap. Note: the AO ratio is never undefined because both F_T1_ and F_T2_ >0. However, if F_T1_+F_T2_ >1, the minimum AO ratio cannot be zero because it is impossible for the selected pixels for each signal to overlap less than the sum of F_T1_+F_T2_–1. The maximum AO ratio also depends on F_T1_ and F_T2_ (Fig. 1B). Specifically, the maximum occurs when the smaller of the selected fractions completely overlaps the larger.
(3)
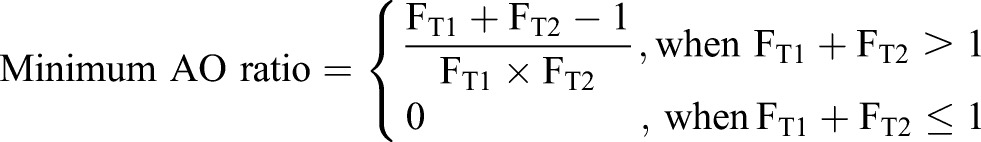

(4)
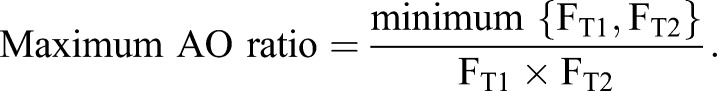
The limits are 0 and 1 for the minimum AO ratio and 1 and +∞ for the maximum AO ratio. When the AO ratio=1, the observed overlap is the same as expected for the null distribution.

The third step is rescaling AO ratios so they can be compared for different thresholds (Fig. 1C). This is necessary because the AO ratio depends on the product of F_T1_ and F_T2_ (see Eqns 2-4). For example, an AO ratio with 100% overlap will be 2 or 10 depending on whether 50% or 10% of the pixels are selected for both signals. Another reason for rescaling is the inherent asymmetry of ratios. Quadrupling the numerator increases the AO ratio from 1 to 4 while quadrupling the denominator decreases it from 1 to 1/4; the latter is a much smaller absolute change. One approach to rescaling is to logarithmically transform the data, which has some advantages (see Supplementary information) but the rescaled values are not easily interpreted. We found that a simple linear rescaling also works well and the results are far easier to interpret.

Linear rescaling generates a metric called the threshold overlap score (TOS). TOS rescales the AO ratios so they have a range from -1 to +1 for all thresholds and have a value of 0 when the observed overlap is exactly the same as expected for the null distribution (Fig. 1D).
(5)
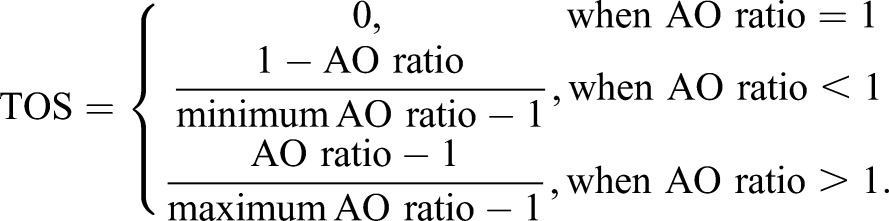
The magnitude of TOS reflects how much the overlap lies between the null hypothesis and the maximum or minimum values; for example an absolute value of TOS=0.9 is nine tenths from the null distribution to the maximum or minimum possible overlap.

It is helpful to divide the spectrum of possible TOS values into categories of ‘colocalization’, ‘anti-colocalization’ and ‘non-colocalization’. In doing so, it is important to recognize that TOS values may be too small to be biologically relevant (Martinez-Abrain, 2008; Lovell, 2013) even if they show statistically significant differences from the null distribution. It is also not useful to define non-colocalization as exactly equal to zero because very few samples would be in this category. For these reasons, we recommend defining non-colocalization as a range of values, e.g. TOS between -0.1 and +0.1. A practical advantage of defining non-colocalization as a range is that a ‘true’ non-colocalized pattern can be consistently referred to as such, rather than as ‘weak colocalization’ in one measurement and ‘weak anti-colocalization’ in another due to measurement error and randomness in biological variation. It must be stressed that these bounds are for the convenience of interpretation and do not affect the analysis itself, and that the definition of non-colocalization should be guided by the design and purpose of the study.

### Generating TOS matrices

One of the most difficult aspects of measuring localization is selecting the thresholds (Bolte and Cordelieres, 2006). Thresholds can affect the contribution to the analysis of background signals from the imaging system (if it is not subtracted) and from cells (e.g. autofluorescence) and low intensity signals from unbound or non-specifically bound fluorescent, chemiluminescent or colorimetric probes or stains. These low intensity signals, which we refer to as non-specific or ‘off-target’ signals, typically have higher intensity than background signal but lower intensity than ‘on-target’ signals where the probe or stain has localized to the biological target. Separating background and off-target signals is often difficult, and it is typically more important to distinguish both of them from on-target signals. Even if there is no background and off-target signals, threshold selection can affect quantification of localization. Therefore protocols have been developed to make threshold selection less arbitrary (Bolte and Cordelieres, 2006; Costes et al., 2004) but they do not always function well, especially when on-target signals are anticorrelated or the background and off-target signals are high or correlated (Dunn et al., 2011). Furthermore, cells often have mixed localization patterns making the evaluation of localization at a single set of thresholds, no matter how they are chosen, an inaccurate ensemble description of localization. Based on all the above, we chose to systematically calculate TOS at many different thresholds resulting in a TOS matrix, which can be viewed as a heat map (Fig. 1E). As will be shown below, the TOS matrix can reveal trends between localization and signal intensity, and allow the identification of multiple localization patterns within cells and organisms.

A TOS matrix can in theory be generated by taking any number of combinations of thresholds for each channel, ranging from a single set of thresholds to a set of thresholds from every different pixel intensity value in a cell. The former would create a matrix with one element and the latter would create a matrix with up to N^2^ elements, where N is the total number of pixels in the cell. While having the maximum number of combinations will give maximum resolution, creating it for every cell would be problematic for many reasons including: (i) being too computationally intensive; and (ii) resulting in different sized TOS matrices for each cell (because they have different numbers of pixels), which makes it harder to combine them (see below). It must also be kept in mind that if thresholds are taken at the very highest and lowest selected fractions there may be too few overlapping or non-overlapping pixels respectively for statistical significance unless large numbers of cells are measured. Note: *a priori* statistical power can be estimated with standard parametric tests and then increased by up to 15% to account for post hoc non-parametric tests having less power (Hodges and Lehmann, 1956; Clifford Blair et al., 1980). While the highest thresholds will select a lower number of pixels, these pixels will have the highest numbers of reporter molecules (hence the higher intensity signal) and thus tend to have a lower coefficient of variation.

We chose an intermediate number of threshold combinations (specifically 81 combinations) and found that it gave more than adequate resolution to detect different patterns of localization in our simulations and the experimental data we analyzed (see below). These threshold combinations were nine selected fractions for signals 1 and 2 (F_T1_ and F_T2_) from 0.9 to 0.1 in increments of 0.1. Initially, 10% of pixels with the lowest intensity pixels are removed from both signals (leaving a selected fraction of 90% of the pixels; i.e. F_T1_ and F_T2_=0.9), then 20% of the lowest intensity pixels in the entire cell are removed for one or both signals (leaving a selected fraction of 80% for signal 1 or 2), and so on, until 90% of the lowest intensity pixels in the entire cell are removed for one or both signals (leaving a selected fraction of 10% for signal 1 and 2). Note: F_T1_ and F_T2_=1 were not included in the analysis because these selected fractions correspond to 100% of the pixels in the cell therefore all selected pixels must overlap and TOS=0.

It can be necessary and convenient to extract values from TOS matrices that quantify specific features of mixed localization patterns, and three values that were found to be especially useful were (see below): (i) TOS at the highest thresholds (TOS_h_), which correspond to the lowest selected fractions (Fig. 1F); (ii) the maximum TOS in the matrix (TOS_max_), which if >0 specifies thresholds with maximum colocalization; and (iii) the minimum TOS in the matrix (TOS_min_), which if <0 specifies thresholds with maximum anti-colocalization. TOS_h_ was chosen because many analyses will want to specifically measure the localization pattern of the on-target signal, which will usually be most separated from any background and off-target signals at the highest intensity values (see data below). If the localization pattern is similar at all intensities or the localization pattern of the pixels with the highest signal intensity is not reflective of the biology (including signal due to noise) then a lower threshold should be selected. Other criteria could also be used to select values from the TOS matrix (see Discussion) and their selection should be guided by the experimental system, biological questions, and the heterogeneity of the data.

### Interpretation of TOS analysis in samples with mixed localization patterns

**Fig. 2.**
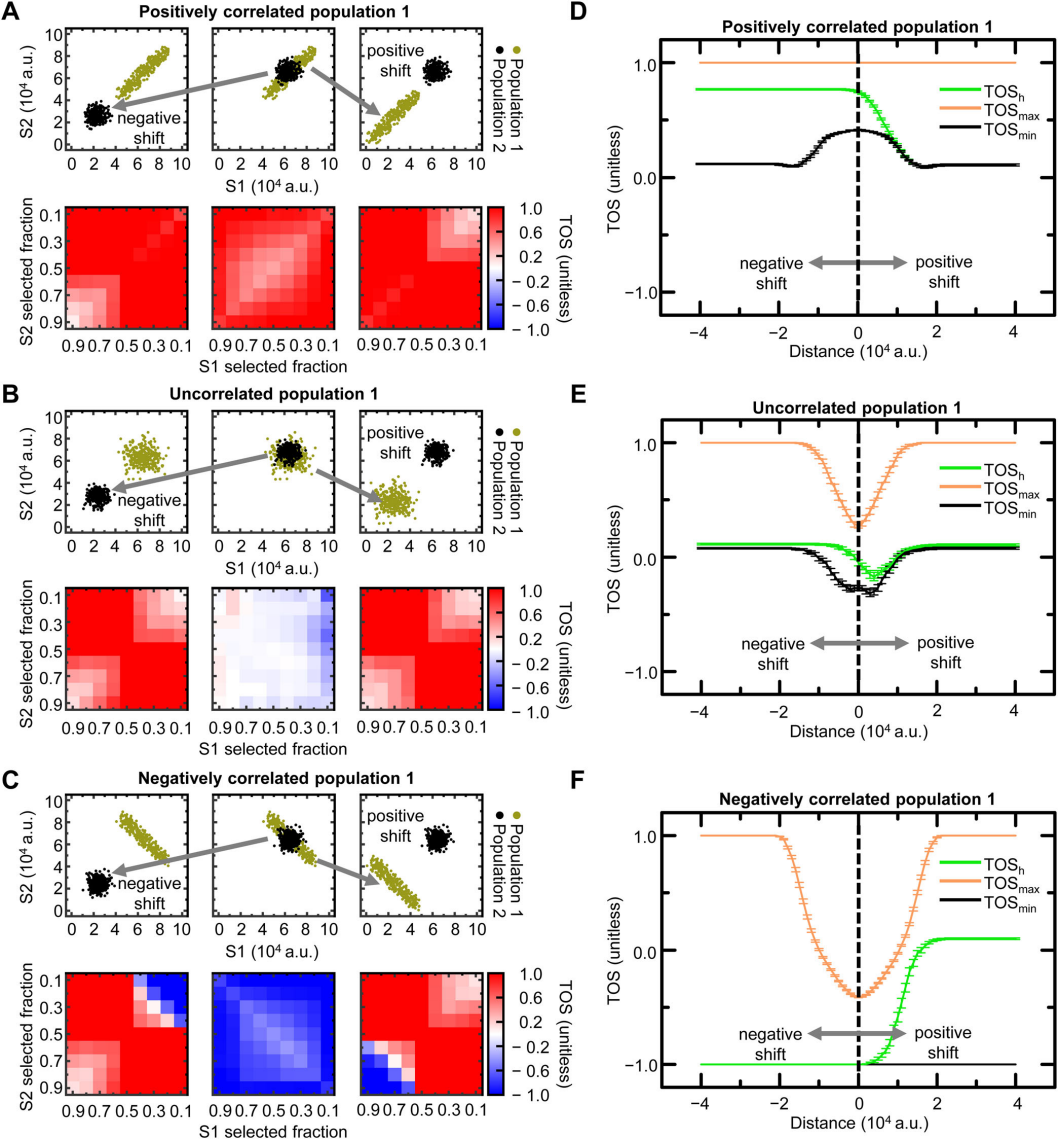
**Interpretation of TOS analysis in samples with mixed localization patterns.** (A-C) Scatterplots and their corresponding TOS matrices for images with two populations of pixels (*n*=300 for each population). Population 1 was positively correlated (A), uncorrelated (B), or negatively correlated (C), and population 2 was always uncorrelated. The means of the two populations were initially the same (center column) and either population 2 was decreased (left column) or population 1 was decreased (right column). Decreasing population 1 and decreasing population 2 corresponds to the positive and negative distances respectively in panels D-F. Populations 1 and 2 were decreased in 40 increments. Only scatterplots and TOS matrices for the initial and final positions are shown. (D-F) Mean TOS_h_, TOS_max_ and TOS_min_ at each distance of the populations (total=81). Panels D, E and F correspond to distributions where population 1 is positively correlated, uncorrelated, or negatively correlated. Parameters were calculated from 50 TOS matrices simulated for each distance. Error bars are the standard error of the mean (s.e.m.). Note: in panel F, the line for TOS_min_ is covered by the line for TOS_h_ at distances <0 a.u.

We simulated cells to demonstrate how TOS matrices can appear with mixed patterns of localization. The simulated cells had two subpopulations of pixels, which for simplicity had equal counts and uniformly distributed random noise [range 0 to 1×10^4^ arbitrary units (a.u.)]. Population 1 was either positively correlated, uncorrelated, or negatively correlated for signals 1 and 2 (scatterplots, Fig. 2A-C) and population 2 always had uncorrelated signals 1 and 2 (scatterplots, Fig. 2A-C). The two populations initially overlay one another (mean=6.5×10^4^ a.u. for both signals; middle scatterplots in Fig. 2A-C). The mean of population 1 or 2 was decreased in 40 equal increments [until the mean=2.5×10^4^ a.u. (left and right scatterplots, Fig. 2A-C)]. The population with the lower intensity signal can be considered to represent background and off-target signals and the population with the higher intensity signals can represent the on-target signal. Note: the absolute values and units of pixel intensity are not important because the pixels are rank ordered according to intensity and thresholds are a selected fraction of the pixels rather than values.

For every cell we calculated a TOS matrix. But only those cells where populations 1 and 2 are at their lowest and highest mean intensities (lower left and lower right in Fig. 2A-C), and where populations 1 and 2 maximally overlay each other (lower center in Fig. 2A-C) are displayed as heat maps. Visual inspection of the TOS matrices shows the upper right corner accurately reflects whether pixels with the highest intensities are colocalized (positively correlated), non-colocalized (uncorrelated) or anti-colocalized (negatively correlated). Similarly, the lower left corner of the TOS matrices reflects whether pixels with the lowest intensities are colocalized, non-colocalized or anti-colocalized, even though the analysis also includes pixels with high intensities.

We extracted TOS_h_, TOS_max_ and TOS_min_ from the TOS matrices and plotted them as a function of the distance between populations 1 and 2 (with negative and positive shifts indicating the means of signals 1 and 2 for population 2 are less and more respectively than population 1) (Fig. 2D-F). TOS_h_ correctly shows when the population with the higher means was colocalized, anti-colocalized or non-colocalized (i.e. TOS_h_ >0.1, ∼0 and <-0.1 respectively) (Fig. 2D-F). TOS_max_ identified when the combined population (i.e. populations 1 and 2 considered together) displayed colocalization but was insensitive to the localization patterns of each subpopulation. TOS_min_ was sensitive to the presence of anti-colocalization in population 1 irrespective of whether it had the lower or higher mean of the two populations (scatterplots in Fig. 2C and black line in Fig. 2F). Values for TOS_h_, TOS_max_ and TOS_min_ were found to be relatively insensitive to the mean intensity and distance between the populations; that is, they provide a robust measure of localization.

In summary, the TOS matrix is helpful in distinguishing colocalization, anti-colocalization, and non-colocalization within mixed patterns of localization. TOS_h_, TOS_max_ and TOS_min_ from the TOS matrices appear to be robust measures of: (i) the localization pattern of the on-target signal, (ii) colocalization for all signals together (background, off-target and on-target), and (iii) the presence of anti-colocalization within a mixed localization pattern.

### Comparison of TOS with other metrics of localization

We next compared TOS_h_, TOS_max_ and TOS_min_ to common metrics for evaluating localization using simulated cells with mixed localization patterns. It is not feasible to compare all the alternative metrics to TOS (Bolte and Cordelieres, 2006; Dunn et al., 2011; Lagache et al., 2015; Zinchuk et al., 2007). Therefore, we selected three metrics that are commonly used by experimentalists, which are Pearson's correlation coefficient (PCC), Manders' colocalization coefficients M1 and M2, and Spearman's rank correlation coefficient (SRCC).

PCC calculates the linear correlation in the intensity of two signals (Dunn et al., 2011). SRCC evaluates whether the rank order of values for two signals is the same or not and it does not matter whether this monotonic relationship is linear or nonlinear. M1 and M2 are calculated from the sum of the intensities of the pixels that exceed the thresholds for both signals 1 and 2 divided by the sum of the intensities of the pixels that exceed the threshold for signals 1 or 2, respectively (Manders et al., 1993; Dunn et al., 2011). M1 and M2 depend on the fraction of overlapping pixels, the intensities of the pixels, and the thresholds. It has been proposed that the ‘expected’ M1 and M2 (which are equivalent to F_T2_ and F_T1_) should be subtracted from the observed M1 and M2 respectively, resulting in ‘M1diff’ and ‘M2diff’ (McDonald and Dunn, 2013). Thresholds for M1 and M2 (and consequently for M1diff and M2diff) are commonly selected using a method (or a variant of it) described by Costes and colleagues (Bolte and Cordelieres, 2006; Costes et al., 2004). Costes' method evaluates the correlation in pixels below each threshold in the data, and then selects the threshold with the minimum correlation or highest threshold with a non-positive correlation. Note: we used the former from JACoP (Bolte and Cordelieres, 2006).

**Fig. 3.**
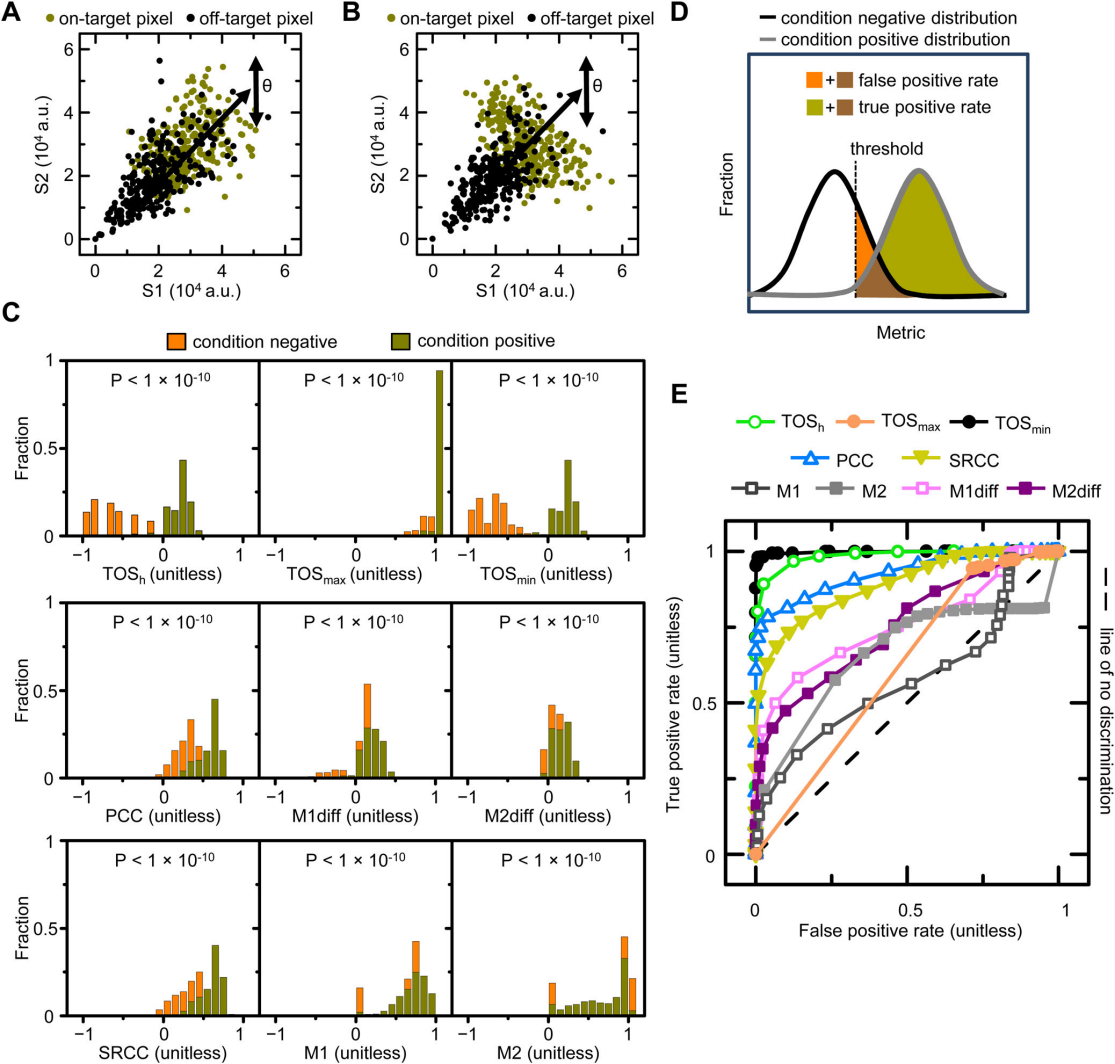
**Comparison of TOS with other metrics of localization.** (A,B) Representative scatterplots for simulated condition positive (A) and condition negative (B) cell populations. All cells have positively correlated off-target signal (black symbols) with a slope (θ) that was varied (see main text). Condition positive cells have on-target signal (gold symbols) that is positively correlated and condition negative cells have on-target signal that is negatively correlated. Both conditions have an equal number of off-target and on-target pixels. (C) Histograms of TOS_h_, TOS_max_, TOS_min_, PCC, SRCC, M1, M2, M1diff, and M2diff for the simulated condition positive and negative populations. *P*-values are calculated using a two-tailed Mann–Whitney U test. Note: M2 values appear to exceed 1 because values that are exactly equal to 1 are in the 1 to 1.1 bin. (D) Diagram explaining the calculation of the true positive and false negative rates for the metrics in panel C (see main text). The fraction of cells in the condition positive population and condition negative population that are above the threshold are the true positive rate and true negative rate respectively. (E) Receiver operating characteristic (ROC) curves for each metric (see main text).

We examined how all metrics performed at distinguishing populations of cells with mixed localization patterns for off-target and on-target signals; this is a challenging, common and important scenario in localization analysis. The metrics were compared using receiver operating characteristic (ROC) curves, which are commonly used to evaluate image analysis tools and diagnostic tests (Metz, 2006). To create the ROC curves, we first simulated ‘condition positive’ and ‘condition negative’ populations of cells with mixed localization patterns (Fig. 3A,B). Each condition positive cell had an equal combination of pixels with positively correlated off-target signal and positively correlated on-target signal (means=20,000 and 30,000 a.u. respectively; total pixels per cell=600). The values were multiplied by a random number from a Gaussian distribution (mean=1.0 and σ=0.2), which was independent for each channel and pixel (Fig. 3A). Each cell in the condition negative population was generated in the same manner except the on-target signal was anticorrelated (Fig. 3B). In the condition positive and negative populations the off-target signal had a slope of θ=e^q^, where q had one of 141 equal increments in the range −0.7 to +0.7. Note: the off-target signal was chosen to be positively correlated (rather than uncorrelated or negatively correlated) because this is often harder to threshold and discriminate from on-target signal, and we sought to compare the metrics under challenging conditions. For each slope, 50 cells were simulated resulting in 7050 cells for each condition.

We evaluated TOS_h_, TOS_max_, TOS_min_, PCC, SRCC, M1, M2, M1diff and M2diff for each cell. For every metric, *P*-values were calculated using the two-tailed Mann–Whitney U test which showed all metrics had statistically significant differences in their values for the condition positive and negative populations (displayed in Fig. 3C). Therefore a simple statistical comparison is not helpful in comparing the metrics. Histograms of the values for the condition positive and negative populations were generated and then the fractions of cells in each population above a threshold that slides from highest to lowest value were determined. These fractions for the condition positive and negative populations are the true positive rates (also known as the sensitivity) and false positive rates (which is 1−specificity) respectively (Fig. 3D). The true positive rates were plotted as a function of the false positive rates at each threshold to produce ROC curves for each metric (Fig. 3E). The ROC curve nearest the upper left corner of the plot is closest to an ideal test with perfect classification of localization, i.e. 100% sensitivity and 100% specificity.

For the simulations, TOS_min_ was the best classifier followed by TOS_h_ (Fig. 3E). TOS_max_ did not perform well because there were positive correlations at high selected fractions for both the condition positive and condition negative populations, therefore it could not distinguish them (Fig. 3A,B). Similarly, PCC was generally positive for both populations, which is why it did not perform as well as TOS_h_. M1diff and M2diff did not perform well due to Costes' method for threshold selection (note: also in some cases a threshold could not be identified resulting in undefined M1diff, and M2diff that were not included in the analyses). Consistent with the study mentioned above (McDonald and Dunn, 2013), M1diff and M2diff performed better than M1 and M2, therefore the former two were used in subsequent analyses.

### Application of TOS to different types of experimental data

We demonstrated the generality of TOS analysis by calculating matrices and extracting TOS_h_, TOS_max_, and TOS_min_ for experiments with different proteins in a variety of cells and organisms, which were obtained from public image repositories (Materials and Methods).

**Fig. 4.**
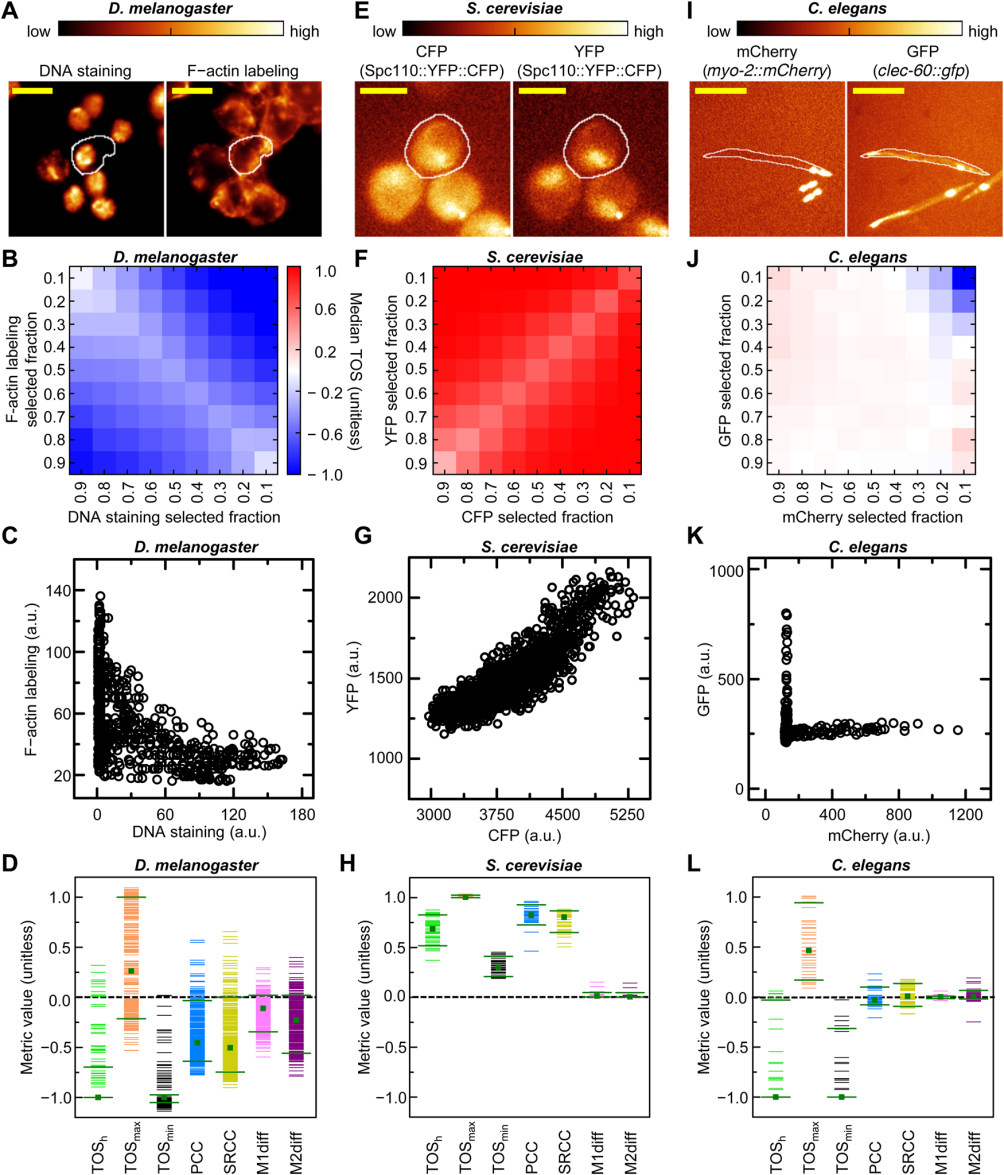
**Application of TOS to different types of experimental data.** (A) Microscopy images of representative *D. melanogaster* cells with DNA staining (Hoechst 33342) and F-actin labeling (fluorescein-conjugated phalloidin). Images are pseudocolored and white lines indicate cell boundaries. Scale bar: ∼5 µm (Velichkova et al., 2010; George et al., 2014). (B) TOS matrix analysis in *D. melanogaster* cells (*n*=366) with selected fractions for DNA staining and F-actin labeling intensities. (C) Scatterplot of DNA staining and F-actin labeling in the outlined *D. melanogaster* cell in panel A. Note: all intensity values are >0. (D) TOS_h_, TOS_max_, TOS_min_, PCC, SRCC, M1diff, and M2diff values obtained in individual *D. melanogaster*. Green lines and square indicate the 90th and 10th percentiles and the medians. Horizontal dash line at zero indicates non-colocalization or no correlation. (E) Microscopy images of representative *S. cerevisiae* cells with Spc110::YFP::CFP. Images presented as in panel A are pseudocolored. Scale bar: 5 µm (obtained from YRC PIR image). (F) TOS matrix analysis in *S. cerevisiae* cells (*n*=38) with selected fractions for CFP and YFP fluorescence intensity. Heat map scale shown in panel B. (G) Scatterplot of CFP and YFP fluorescence in the outlined *S. cerevisiae* cell in panel E. (H) TOS_h_, TOS_max_, TOS_min_, PCC, SRCC, M1diff, and M2diff values obtained in individual *S. cerevisiae*. Data presented as in panel D. (I) Microscopy images of representative *C. elegans* with mCherry and GFP fluorescence. Scale bar: ≈500 µm (Wahlby et al., 2012). Images presented as in panel A except the white line is a *C. elegans* outline. (J) TOS matrix analysis in *C. elegans* (*n*=42) with selected fractions for mCherry and GFP fluorescence intensity. Heat map scale shown in panel B. (K) Scatterplot of mCherry and GFP fluorescence signal in the *C. elegans* outlined in panel I. (L) TOS_h_, TOS_max_, TOS_min_, PCC, SRCC, M1diff, and M2diff values obtained in individual *C. elegans*. Data presented as in panel D.

The first dataset examined were *Drosophila melanogaster* Kc167 cells (*n*=366), which had been probed with fluorescein-conjugated phalloidin to identify F-actin in the cytoskeleton and stained with Hoechst 33342 to identify DNA (Wheeler et al., 2004; Carpenter et al., 2006) (Fig. 4A). TOS matrices from individual cells were combined and the median TOS value for each threshold combination was presented as a heat map (Fig. 4B). This analysis shows that at most selected fractions the F-actin probe and DNA staining are strongly anti-colocalized (i.e. TOS <<0), which is expected because they label different parts of the cell, i.e. outside the nucleus and in the nucleus, respectively. Scatterplots also show anti-colocalization with the intensities of F-actin labeling and DNA staining being largely independent (Fig. 4C). TOS_h_, TOS_max_, and TOS_min_ from individual cell TOS matrices were compared to PCC, SRCC, M1diff and M2diff from the same cells. The values of each metric for individual cells were plotted along with the median and 90th and 10th percentile values. Note: zero indicates no correlation or non-colocalization (Fig. 4D). In >90% of cells, TOS_h_ and TOS_min_ indicate anti-colocalization and their medians are ≈−1, i.e. maximally anti-colocalized. In contrast, TOS_max_, PCC, SRCC, M1diff and M2diff have many values between the 90th and 10th percentiles that are close to or greater than zero, which indicates these metrics classify many cells as having non-colocalization or colocalization rather than the expected anti-colocalization (Fig. 4D).

The second dataset examined were *Saccharomyces cerevisiae* cells (*n*=38), which have a single copy of the spindle pole body component protein (Spc110) fused to both yellow fluorescent protein (YFP) and cyan fluorescent protein (CFP), i.e. Spc110::YFP::CFP (Muller et al., 2005). We used eight sets of images (YRC PIR ID: 191, 3208, 3559, 3702, 3999, 4722, 5160, and 7396) with YFP, CFP and differential interference contrast (DIC) channels (Fig. 4E). The analysis was performed as described for *D. melanogaster*. Because YFP and CFP are part of the same protein their signals should colocalize, and this was clearly seen at all threshold levels in the TOS matrix and scatterplot (Fig. 4F,G). TOS_h_, TOS_max_, TOS_min_, PCC and SRCC correctly identified that >90% of cells have strong colocalization (Fig. 4H). M1diff and M2diff incorrectly identified most cells as non-colocalized (Fig. 4H) due to Costes' method selecting very low thresholds; and this in turn results in the subtraction of a large ‘null’ value and therefore M1diff and M2diff are small. Note: this is one reason the rescaling for TOS is so helpful.

The third dataset examined were *Caenorhabditis elegans* (*n*=42) which had the production of green fluorescent protein (GFP) regulated by the activity of the *clec-60* promoter (Fig. 4I). In the *pmk-1* deficient mutant that was imaged, GFP production is increased in the anterior intestine next to the pharynx which was identified by the mCherry fluorescent protein transcribed under the control of the *myo-2* gene (Wahlby et al., 2012) (Fig. 4I). The analysis was performed as for the other datasets, and a matrix of TOS median values showed anti-colocalization of the GFP and mCherry fluorescence signals at low selected fractions, i.e. high threshold (upper right corner, Fig. 4J). Anti-colocalization is both consistent with the biology (because GFP and mCherry label different structures) and a scatterplot of a representative *C. elegans* (Fig. 4K). Values of TOS_h_ and TOS_min_ indicated anti-colocalization in >90% of worms (both green horizontal bars are below zero in Fig. 4L). Most TOS_max_ values were >0 therefore it was possible in most worms to identify a set of thresholds where there was a colocalization pattern, which was typically when the selected fraction was high in one channel and low in the other channel. In contrast to TOS_h_ and TOS_min_, values for PCC, SRCC, M1diff and M2diff were generally around zero indicating non-colocalization for most worms (Fig. 4L). The latter group of metrics performed poorly because it was difficult to identify a threshold that distinguishes the overlapping off-target and on-target signals and Costes' method tended to choose high selected fractions, i.e. low threshold values.

In summary, TOS analysis successfully identified the expected localization patterns of different proteins in various cells and organisms. In these images, TOS_h_, TOS_max_, or TOS_min_ were often able to identify specific features within mixed localization patterns better than PCC, SRCC, M1diff and M2diff.

### TOS values can distinguish localization patterns in experimental data with high specificity and high sensitivity

**Fig. 5.**
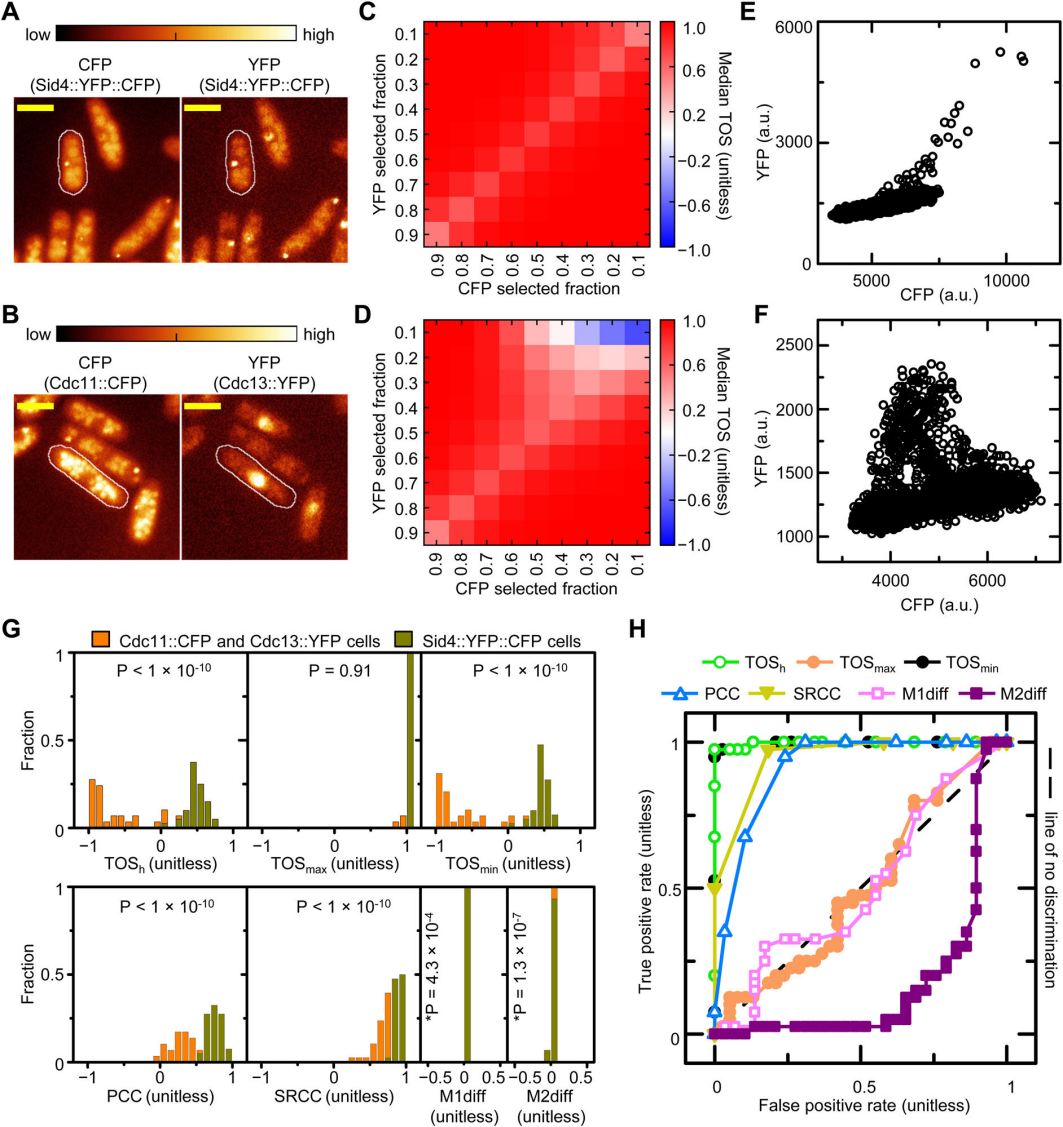
**TOS values can distinguish localization patterns in experimental data with high specificity and high sensitivity.** (A) Microscopy images of representative *S. pombe* cells with Sid4::YFP::CFP. Images are pseudocolored. White lines indicate cell boundaries. Scale bar: 10 µm (obtained from YRC PIR images). (B) Microscopy images of representative *S. pombe* cells with Cdc11::CFP and Cdc13::YFP. Images are presented as in panel A. (C,D) TOS matrices for the strains in panels A and B, respectively (*n*=40 and 38). Each matrix shows the median values obtained for TOS matrices of individual cells. (E,F) Scatterplots of CFP and YFP signal intensities for the outlined cell of each strain in panel A and B, respectively. (G) Histograms of TOS_h_, TOS_max_, TOS_min_, PCC, SRCC, M1diff and M2diff values for individual cells in each strain. *P-value is calculated with the raw data, which has more variation than seen in the binned data of the histogram. (H) ROC curves for all metrics in panel G.

We investigated how well TOS and other metrics can distinguish similar localization patterns. Two types of *Schizosaccharomyces pombe* strains were chosen with fluorescent proteins that were expected to show colocalization. One strain (*n*=40) had the fusion protein Sid4::YFP::CFP (strain KG4608; ID: 192, 776, 1062 and 1233) (Fig. 5A). Because these fluorescent proteins are fused they should colocalize. A second strain (*n*=38) had two fusion proteins: Cdc11::CFP and Cdc13::YFP (strain KG3544; ID: 292, 360, 414, and 744) (Fig. 5B). Cdc11::CFP and Cdc13::YFP are known to localize to the spindle pole body (Krapp et al., 2001; Decottignies et al., 2001) as well as to other sites.

Cells from each strain were identified and analyzed as described above. TOS matrices for both strains showed colocalization (TOS >>0) at many threshold combinations (Fig. 5C,D). However, at high signal intensities, i.e. small F_T1_ and F_T2_, there are differences in localization between the two strains; cells with Sid4::YFP::CFP have colocalization and cells with Cdc11::CFP and Cdc13::YFP have anti-colocalization (Fig. 5C,D). The difference in localization can also be seen in the scatterplot of CFP and YFP signal intensity in a representative cell from each strain (Fig. 5E,F). That is, pixels with high intensity CFP and YFP signals tend to occupy the upper right corner for Sid4::YFP::CFP but tend to be at the right or at the top for Cdc11::CFP and Cdc13::YFP. Note: there are many possible causes for why there is more YFP fluorescence for a given amount of CFP fluorescence at high intensity levels compared to lower intensity levels for the YFP::CFP fusion (Fig. 5E) including: increased transcription and translation termination, and decreased CFP fluorescence and/or increased YFP fluorescence due to altered protein folding and aggregate formation.

TOS_h_, TOS_max_, TOS_min_, PCC, SRCC, M1diff and M2diff were calculated as for the analysis above (Fig. 3D). All metrics except TOS_max_ had statistically significant differences in the distribution of values for the two strains (displayed in Fig. 5G). To compare metrics we generated histograms of each value from individual cells (Fig. 5G) and then ROC curves (Fig. 5H) as described above. Cells with Sid4::YFP::CFP were designated the condition positive population and cells with Cdc11::CFP and Cdc13::YFP were the condition negative population. ROC curves for TOS_h_ and TOS_min_ demonstrated that they can discriminate the localization patterns of the two cell types with greater specificity and sensitivity than SRCC, PCC, M1diff and M2diff. M1diff and M2diff were not able to distinguish the localization patterns in the two strains because of difficulty with thresholding.

In summary, values from TOS matrices such as TOS_h_ and TOS_min_ were able to distinguish the localization patterns of the different proteins with greater specificity and greater sensitivity than the other common metrics examined, and they are particularly useful when there are mixed patterns of localization within each cell and thus measures of the localization pattern of entire cells are less meaningful. We stress that this assessment did *not* evaluate whether TOS_h_, TOS_max_, TOS_min_ are better descriptors of an entire population of pixels or whether they identify a specific feature that is reflective of the underlying biology.

## DISCUSSION

Measuring localization is a basic requirement of cell biology and imaging and yet it is often a challenging task. This study demonstrates the TOS metric and its application, and shows that TOS is a valuable tool to help meet the challenge of quantifying localization in a wide range of applications.

TOS has many features that make it suitable for general applications. The first feature is that TOS is simple to interpret because it only quantifies whether signal overlap is the same, more, or less than expected by chance. In contrast, some overlap metrics have a weighting for signal intensities, e.g. Manders' coefficients M1 and M2, ‘overlap coefficient’, and ‘k_1_ and k_2_ coefficients’ (Cordelieres and Bolte, 2014), which means that any value is an unknown combination of two factors: overlap and intensities. A second feature is that TOS can be compared at different thresholds, which is not the case with some other metrics, e.g. M1 and M2 coefficients (McDonald and Dunn, 2013). A third feature is that a single value distinguishes between colocalization, anti-colocalization and non-colocalization, whereas some metrics require two values for interpretation, and/or they do not directly distinguish between anti-colocalization and non-colocalization (Cordelieres and Bolte, 2014). A fourth feature is the null hypothesis for TOS has minimal assumptions and requires no simulations (McDonald and Dunn, 2013; Bolte and Cordelieres, 2006), which makes it easier to implement. A fifth feature is that TOS is one of the metrics that does not assume a linear correlation in signal intensities (Bolte and Cordelieres, 2006).

The general applicability of TOS is enhanced by systematically evaluating it at many different threshold combinations. The resulting TOS matrix is particularly useful when there are mixed patterns of localization; and the background and off-target signals are continuous with the on-target signal. TOS matrices are best interpreted holistically with TOS at each selected fraction being evaluated in the context of neighboring TOS (which can detect trends and provide confidence for a specific value for TOS) and in relation to other localization patterns found in the matrix. Within TOS matrices, TOS at the highest thresholds (i.e. TOS_h_) was particularly helpful in identifying localization patterns in on-target signals when the off-target and background signals were at high levels and/or occupying a large proportion of pixels. We showed that TOS_h_, as well as TOS_max_ and TOS_min_, can have greater specificity and sensitivity than PCC, SRCC, M1diff, and M2diff. Furthermore, TOS was very easy to use with a wide variety of proteins, cell types, and organisms. For all the above reasons, TOS matrices are a good first line of analysis for quantifying intracellular localization. However, we reiterate that there is no best test for all situations (Bolte and Cordelieres, 2006; Dunn et al., 2011) and that the selection of a metric must take into account the purpose of the analysis, the underlying biology, and the types of images and samples.

To interpret values of TOS it is important to note that in many imaging experiments, including those used in this study, the concentration of reporter is high and single particles cannot be resolved. Therefore the signal in each pixel (or voxel) is the total of many reporter molecules within an area (or volume) of the cell. That is, the signal intensity in each pixel reflects the local concentration of a molecule. Local concentrations may be higher or lower in some cell regions depending on: (i) sites of production and degradation; (ii) diffusion; (iii) kinetics of association and dissociation with cellular structures, e.g. nucleus, cell membrane or cytoskeleton; and (iv) attraction to or exclusion from cell regions (Neeli-Venkata et al., 2016; Mondal et al., 2011). With this in mind, colocalization, anti-colocalization and non-colocalization should be considered as the relationship in the local concentrations of two types of molecules, which may be due to many factors. Therefore colocalization should not by itself be interpreted as indicating that two types of molecules are bound to each other (Bolte and Cordelieres, 2006; Dunn et al., 2011). Note: the above mechanisms could potentially generate concentration gradients that contribute more to the spatial autocorrelation of signals in cells than point spread functions (Dunn et al., 2011; Xu et al., 2016).

Following from the above, colocalization indicates that higher concentrations of two molecules tend to occur in similar cell regions. This may be due to common sites of production, action, binding, or degradation. Anti-colocalization indicates two molecules have high concentrations in different cell regions and thus at least one mechanism is causing the molecules to be recruited to and/or exclude from different regions, one molecule excludes the other from a region (Bakshi et al., 2015; Neeli-Venkata et al., 2016) or the molecules eliminate each other in the same location, e.g. when non-coding RNAs binding to mRNAs both are destroyed (De Lay et al., 2013). Non-colocalization indicates that molecules have no preference for avoiding or occurring in the same regions. Because the mechanisms responsible for generating anti-colocalization and non-colocalization are different, the capacity of metric such as TOS to distinguish these patterns is potentially very useful.

The TOS metric could be adapted for applications that were not examined in this study and to measure localization in different ways. We chose to measure overlap by selecting pixels above thresholds because that approach was most similar to that of Manders' colocalization coefficients. However, AO, AO ratio and TOS could be modified to measure the overlap of pixels below a threshold or within a range, i.e. the equivalent of a band-pass filter or low-pass filter instead of a high-pass filter. Another way in which the TOS metric could be altered is to choose selected fractions of pixels by features other than signal intensity such as their distances to the cell poles or membrane. Additionally, TOS analysis could be adapted to examine localization in three dimensional images, e.g. images assembled from confocal microscopy, or measure the overlap of more than two signals.

In conclusion, systematic evaluation of the TOS metric at multiple threshold combinations is a valuable addition to the repertoire of tools available for the quantitative analysis of images. TOS analysis is simple to implement and easy to interpret, and it has many features that make it suitable for many types of images and samples. Furthermore, values from TOS matrices can distinguish patterns of localization with greater sensitivity and greater specificity than other commonly used metrics. These findings make a strong case for selecting TOS analysis as a first step to evaluating localization in images.

## MATERIALS AND METHODS

### Simulations, calculation of metrics, and statistical analyses

Simulations, calculations and statistical analyses were performed as described in the Results using Matlab (R2015a, Mathworks) (code archived at Figshare: https://figshare.com/s/6504f19aef88f1d6cf95). Post-measurement statistical comparisons were performed using the two-tailed Mann–Whitney U test. Note: localization in one set of samples could also be compared to the median of the expected null distribution using the Sign test or by bootstrapping.

### Receiver operating characteristic (ROC) curves

Histograms for simulated data were generated using the histcounts function in Matlab. This function, which is based on Scott's rule (Scott, 1979), determined the bin edges in the range of −1.2 to 1.2. For experimental data, bin edges had increments of 0.1 for TOS_h_ and TOS_min_ (defined in Results section), Pearson's correlation coefficient, and Spearman's rank correlation coefficient, and increments of 0.001 for the other metrics. The bin edges were used as thresholds and the fraction of counts in each population above the thresholds were used to create the ROC curves.

### Analysis of images

Images of *Drosophila melanogaster* Kc167 cells [BBBC007_v1 (A9)] and whole organism *Caenorhabditis elegans* (BBBC012v1) were obtained from the Broad Bioimage Benchmark Collection (Ljosa et al., 2012). Hand drawn boundaries of *D. melanogaster* cells were downloaded from the same collection and inverted to select cells (Jones et al., 2005). *Saccharomyces cerevisiae* (DHY155) and *Schizosaccharomyces pombe* (KG4608 and KG3544) images were obtained from the Yeast Resource Center Public Image Repository (YRC PIR) (Riffle and Davis, 2010). Boundaries were traced around *C. elegans* in four different brightfield images, around *S. cerevisiae* cells in eight differential interference contrast (DIC) images, and around *S. pombe* in four DIC images for each strain. Traces were performed in ImageJ (Schneider et al., 2012) and these defined the boundary of a ‘region of interest’ (ROI) (data files at Figshare: https://figshare.com/s/e414b6b45d53f79f7b1f). A ‘Count Mask’ was created in ImageJ to fill each ROI in an image with a unique integer. Count Mask was used to select pixels in the fluorescence images with Matlab that correspond to cells or *C. elegans*. Pixel intensity values within each cell or *C. elegans* were stored in an array, which were used for the analyses.

Some downloaded drawn objects for *D. melanogaster* cells did not identify cell boundaries, therefore the Analyze Particle function in ImageJ was used to eliminate small (<400 pixels) and large objects (>5000 pixels) from the analysis. In addition, boundaries that identified areas between cells were eliminated by selecting only ROIs with fluorescence signals greater than the background in non-cell regions. Occasional *S. cerevisiae* cells had binned data so they were removed from the analyses.
